# Supplementary material for: Examining allergy related diseases in Africa: A scoping review protocol
Source: PLoS One. 2024 Feb 20;19(2):e0297949. doi: 10.1371/journal.pone.0297949 (PMC10878524; doi:10.1371/journal.pone.0297949)
Supplement: S1 Appendix — (DOCX) [file pone.0297949.s002.docx]

Supplementary Material

**Title of Review:** Examining allergy related diseases in Africa: A scoping review protocol

**Database:** PubMed
**Platform:** US National Library of Medicine
**Date Searched:** July 11, 2023
**Database Date Coverage:** 1946–2023

**Date Limits:** January 2003–July 2023
**Other Limits:** Human; Excludes: editorials, commentary, conference abstracts, letters, errata, corrigenda, protocols

| **Set** | **Concept** | **Search Strategy** |
| --- | --- | --- |
| #1 | Allergy-Related Diseases | (“atopic dermatit*”[tiab] OR “atopic neurodermatit*”[tiab] OR eczema[tiab] OR asthma[tiab] OR asthmas[tiab] OR asthmatic[tiab] OR “lung allerg*”[tiab] OR “allergic rhinitis”[tiab] OR “allergic rhinopath*”[tiab] OR “atopic rhinitis”[tiab] OR “eosinophilic rhinitis”[tiab] OR “rhinitis allergica”[tiab] OR “pollen allerg*”[tiab] OR “pollen hypersensitiv*”[tiab] OR “Allergic Rhinitides”[tiab] OR “hay fever”[tiab] OR “food allerg*”[tiab] OR “food hypersensitiv*”[tiab] OR “egg allerg*”[tiab] OR “egg hypersensitiv*”[tiab] OR “milk allerg*”[tiab] OR “milk hypersensitiv*”[tiab] OR “nut allerg*”[tiab] OR “nut hypersensitiv*”[tiab] OR “peanut allerg*”[tiab] OR “peanut hypersensitiv*”[tiab] OR “shellfish allerg*”[tiab] OR “shellfish hypersensitiv*”[tiab] OR “fish allerg*”[tiab] OR “fish hypersensitiv*”[tiab] OR “seafood allerg*”[tiab] OR “seafood hypersensitiv*”[tiab] OR “wheat allerg*”[tiab] OR “wheat hypersensitiv*”[tiab] OR “fruit allerg*”[tiab] OR “fruit hypersensitiv*”[tiab] OR “seed allerg*”[tiab] OR “legume allerg*”[tiab] OR “meat allerg*”[tiab] OR "Dermatitis, Atopic"[Mesh] OR "Asthma"[Mesh] OR "Rhinitis, Allergic"[Mesh] OR "Food Hypersensitivity"[Mesh] OR "Egg Hypersensitivity"[Mesh] OR "Milk Hypersensitivity"[Mesh] OR "Nut and Peanut Hypersensitivity"[Mesh] OR "Nut Hypersensitivity"[Mesh] OR "Peanut Hypersensitivity"[Mesh] OR "Shellfish Hypersensitivity"[Mesh] OR "Wheat Hypersensitivity"[Mesh]) |
| #2 | Africa | (Africa[tiab] OR "Africa"[Mesh] OR “African Union”[tiab] OR "African Union"[Mesh] OR "Africa, Northern"[Mesh] OR “north African”[tiab] OR “northern Africa”[tiab] OR "Africa South of the Sahara"[Mesh] OR "Africa, Western"[Mesh] OR “West African”[tiab] OR “western Africa”[tiab] OR “Africa, Southern”[mesh] OR “South African”[tiab] OR “Southern Africa”[tiab] OR “Africa, Eastern”[mesh] OR “Eastern Africa”[tiab] OR “East African”[tiab] OR “Africa, Central”[mesh] OR “Central African”[tiab] OR “Central Africa”[tiab] OR Sahara[tw] OR Saharan[tw] OR subsahara*[tiab] OR Sahel[tw] OR Algeria[tiab] OR Algeria[mesh] OR Angola[tiab] OR Angola[mesh] OR Benin[tiab] OR Benin[mesh] OR Dahomey[tw] OR Botswana[tiab] OR Botswana[mesh] OR “Burkina Faso”[tiab] OR “Burkina Faso”[mesh] OR "Upper Volta"[tw] OR Burundi[tiab] OR Burundi[mesh] OR “Cabo Verde”[tiab] OR “Cabo Verde”[mesh] OR "Canary Islands"[tw] OR "Cape Verde"[tw] OR Cameroon[tiab] OR Cameroon[mesh] OR “Central African Republic”[tiab] OR “Central African Republic”[mesh] OR Chad[tiab] OR Chad[mesh] OR Comoros[tiab] OR Comoros[mesh] OR Congo[tiab] OR Zaire[tw] OR "Democratic Republic of the Congo"[Mesh] OR “Cote d’Ivoire”[tiab] OR “Ivory Coast”[tiab] OR "Cote d'Ivoire"[Mesh] OR Djibouti[tiab] OR Djibouti[mesh] OR Egypt[mesh] OR Egypt[tiab] OR “Equatorial Guinea”[tiab] OR “Equatorial Guinea”[mesh] OR Eritrea[tiab] OR Eritrea[mesh] OR Eswatini[tiab] OR Eswatini[mesh] OR "Swaziland"[tw] OR Ethiopia[tiab] OR Ethiopia[mesh] OR Gabon[tiab] OR Gabon[mesh] OR "Gabonese Republic"[tw] OR Gambia[tiab] OR Gambia[mesh] OR Ghana[tiab] OR Ghana[mesh] OR Guinea[tiab] OR Guinea[mesh] OR “Guinea-Bissau”[tiab] OR "Guinea-Bissau"[Mesh] OR Kenya[tiab] OR Kenya[mesh] OR Lesotho[tiab] OR Lesotho[mesh] OR Liberia[mesh] OR Liberia[tiab] OR Libya[mesh] OR Libya[tiab] OR Madagascar[mesh] OR Madagascar[tiab] OR Malawi[tiab] OR Malawi[mesh] OR Mali[tiab] OR Mali[mesh] OR Mauritania[tiab] OR Mauritania[mesh] OR Mauritius[tiab] OR Mauritius[mesh] OR "Mayotte"[tw] OR Morocco[tiab] OR Morocco[mesh] OR Mozambique[tiab] OR Mozambique[mesh] OR Namibia[tiab] OR Namibia[mesh] OR Niger[tiab] OR Niger[mesh] OR Nigeria[tiab] OR "Saint Helena"[tw] OR "St Helena"[tw] OR "Tristan da Cunha"[tw] OR Nigeria[mesh] OR Rwanda[tiab] OR Rwanda[mesh] OR “Sao Tome”[tiab] OR Principe[tiab] OR "Sao Tome and Principe"[Mesh] OR Senegal[tiab] OR Senegal[mesh] OR Seychelles[tiab] OR Seychelles[mesh] OR “Sierra Leone”[tiab] OR “Sierra Leone”[mesh] OR Somalia[tiab] OR Somalia[mesh] OR “South Africa”[tiab] OR “South Africa”[mesh] OR Sudan[mesh] OR Sudan[tiab] OR Tanzania[tiab] OR Tanzania[mesh] OR Togo[tiab] OR Togo[mesh] OR "Togolese Republic"[tw] OR Tunisia[tiab] OR Tunisia[mesh] OR Uganda[tiab] OR Uganda[mesh] OR Zambia[tiab] OR Zambia[mesh] OR "Rhodesia"[tw] OR Zimbabwe[tiab] OR Zimbabwe[mesh] OR "Sahrawi Arab Democratic Republic"[tw] OR "Somaliland"[tw]) |
| #3 |  | #1 AND #2 |
| #4 | **Limits** | #3 AND ("2003/01/01"[Date - Publication] : "2023/12/31"[Date - Publication]) |
| #5 |  | #4 NOT ("Animals"[Mesh] NOT ("Animals"[Mesh] AND "Humans"[Mesh])) NOT (mice[tiab] OR mouse[tiab] OR murine*[tiab] OR rat[tiab] OR rats[tiab] OR rodent*[tiab] OR dog[tiab] OR dogs[tiab] OR pig[tiab] OR pigs[tiab] OR piglet*[tiab] OR swine[tiab] OR porcine*[tiab] OR animal*[tiab] OR ape[tiab] OR apes[tiab] OR monkey*[tiab] OR gorilla*[tiab] OR chimpanzee*[tiab] OR macaque*[tiab] OR orangutan*[tiab] OR pongo[tiab] OR macaca[tiab] OR "Pan paniscus"[Mesh] OR "Pongo"[Mesh] OR "Macaca"[Mesh] OR "Gorilla gorilla"[Mesh] OR Dogs[mesh] OR Swine[mesh] OR Mice[mesh] OR Rats[mesh] OR "Muridae"[Mesh] OR "Murinae"[Mesh] OR Rodentia[mesh] OR "Models, Animal"[Mesh] OR "Animal Experimentation"[Mesh]) |
| #6 |  | #5 NOT (letter[ptyp] OR editorial[ptyp] OR comment[ptyp] OR news[ptyp] OR "Congress"[Publication Type] OR "Consensus Development Conference"[Publication Type] OR editorial[tiab] OR commentary[tiab] OR “conference abstract*”[tiab] OR “conference proceeding*”[tiab] OR symposium*[tiab] OR "Published Erratum"[Publication Type] OR errata[tiab] OR erratum[tiab] OR corrigenda[tiab] OR corrigendum[tiab] OR protocol[ti] OR protocols[ti]) |

**Notes:** The limits for language (English) and publication year (2003–2023) were applied to the main search using the filters available in PubMed. The keywords were searched in the title and abstract fields in PubMed (i.e., [Title/Abstract]), text field (i.e., [Text Word]), and the controlled vocabulary terms are indicated with [Mesh]. Terms searched in the [Text Word] field are searched in the title, abstract, keywords, and MeSH fields. Phrases were enclosed in quotation marks to force the searching of the exact terms in order presented. To these results, the search strategy to exclude letters, comments, editorials, conference abstracts, protocols, corrigenda, and errata was used. No other limits were applied to the searches.
