# Supplementary material for: Examining allergy related diseases in Africa: A scoping review protocol
Source: PLoS One. 2024 Feb 20;19(2):e0297949. doi: 10.1371/journal.pone.0297949 (PMC10878524; doi:10.1371/journal.pone.0297949)
Supplement: Attachment — Submitted filename: REVIEW REPORT.docx [file pone.0297949.s003.docx]

Thank you for the opportunity to review this article/study protocol. It is interesting and I look forward to reading the findings of the actual study. I have a few minor concerns/clarifications.

1. In some aspects, the writing style appears like the review has already been conducted. For instance, line 123 “Table 1 includes the eligibility criteria used for study selection.”; line 148 “The time frame of the search was limited to the last two decades as to include the most recent and pertinent literature”; line 237 “Similarly, for the purpose of feasibility and managing the scope of the review, studies in which the main findings did not concern allergic diseases were excluded”; etc.
2. I see this statement “The searches will be limited to those published in any language from 2003 to 2023” in line 147-149 to be a repetition of what is already stated in the inclusion/exclusion criteria
3. Line 163-164:” To reduce the risk of bias and errors, each article will be screened by two independent reviewers (MYL, NS, SS, DS, MB)” Do the authors mean four independent reviewers? Because the names in the parentheses are more than two. Or they will do it in pairs?
4. Authors indicate that “During both levels of screening, disagreements will be resolved by the corresponding author (NS)”, line 165. It appears, however, that the correspondent author (NS) is part of the independent reviewers stated in line 164. How then does he/she resolve a disagreement in an unbiased manner?
5. A piece of advice and authors may ignore/disregard it. Assessment of methodological quality of reviewed studies, in most instance, is not part of scoping reviews, but adding this to the review will help improve the overall quality of the study.

Thank you
