# Supplementary material for: Examining allergy related diseases in Africa: A scoping review protocol
Source: PLoS One. 2024 Feb 20;19(2):e0297949. doi: 10.1371/journal.pone.0297949 (PMC10878524; doi:10.1371/journal.pone.0297949)
Supplement: Attachment — Submitted filename: Scoping Review Protocol Rebuttal Letter.docx [file pone.0297949.s004.docx]

To: Pisirai Ndarukwa

Academic Editor, *PLoS ONE*

Dear Dr. Ndarukwa,

Thank you for inviting us to submit a revised version of our manuscript *Examining allergy related diseases in Africa: A scoping review protocol* to *PLoS ONE*. We also thank you for the time and effort both you and the reviewer have put into providing feedback to improve our paper. We have carefully considered the points raised and have made a faithful effort to improve our paper.

To facilitate your review of our revisions, below is a point-by-point response to the concerns/clarification points raised:

1. *In some aspects, the writing style appears like the review has already been conducted. For instance, line 123 “Table 1 includes the eligibility criteria used for study selection.”; line 148 “The time frame of the search was limited to the last two decades as to include the most recent and pertinent literature”; line 237 “Similarly, for the purpose of feasibility and managing the scope of the review, studies in which the main findings did not concern allergic diseases were excluded”; etc.*

Thank you for pointing this out. In these specified instances, we have revised the tense of the writing from past to future. Additionally, in another similar situation in line 243 of the version without track changes (“our scoping review will represent”), the tense has also been revised from past to future.

2. *I see this statement “The searches will be limited to those published in any language from 2003 to 2023” in line 147-149 to be a repetition of what is already stated in the inclusion/exclusion criteria*

We agree that this is repetitive. To most accurately describe our protocol while eliminating this repetition, we have deleted “Published from 2003-2023” from Table 1 regarding the inclusion/exclusion to most accurately describe our methodology. We have opted to remove this description from the inclusion/exclusion criteria table rather than the search strategy section as we will not be voting to include or exclude articles based on publication date during screening. Instead, the search strategy will have effected such a time-restriction.

3. *Line 163-164:” To reduce the risk of bias and errors, each article will be screened by two independent reviewers (MYL, NS, SS, DS, MB)” Do the authors mean four independent reviewers? Because the names in the parentheses are more than two. Or they will do it in pairs?*

We agree that this phrasing can be confusing. We have deleted the initials of the screeners in lines 163-164 and also when describing the data extraction process in line 174 in order to promote clarity, as well as to reflect the fact that additional researchers who are not co-authors on this protocol may aid in screening and data extraction in the final scoping review. Additionally, in line 174, the phrasing has been revised to “by two independent reviewers” to clarify that each study will only be reviewed by two reviewers total.

Similarly, to promote accuracy and clarity regarding our methodology of conflict resolution in data extraction and charting, we have changed lines 175-177 of the track changes edition to read “Any discrepancies in the data will be reviewed and resolved by a separate third reviewer, and if necessary, consensus discussion with the two reviewers.”

4. *Authors indicate that “During both levels of screening, disagreements will be resolved by the corresponding author (NS)”, line 165. It appears, however, that the correspondent author (NS) is part of the independent reviewers stated in line 164. How then does he/she resolve a disagreement in an unbiased manner?*

Thank you for pointing this out. We agree that the corresponding author cannot resolve a disagreement in an unbiased manner if she is one of the independent reviewers who voted on the study in the first place. Thus, we have amended our criteria surrounding conflict resolution. The corresponding author (NS) will still resolve disagreements at the level of title/abstract screening to facilitate ease and feasibility of conflict resolution. However, at the level of full-text screening, disagreements will be resolved via group discussion or an independent, separate third reviewer. This ensures that at the final and most thorough stage of screening to determine inclusion or exclusion of articles, each article will undergo a rigorous and unbiased review including during potential conflict resolutions.

5. *A piece of advice and authors may ignore/disregard it. Assessment of methodological quality of reviewed studies, in most instance, is not part of scoping reviews, but adding this to the review will help improve the overall quality of the study.*

Thank you for this suggestion. We considered conducting assessment of study quality as part of our review, however, given that it ultimately is not an element of most scoping reviews, decided against such a step. We also have already taken some other measures towards ensuring a high quality of included studies such as excluding grey literature.

Thank you again for providing the opportunity for us to strengthen our manuscript and we hope that it is now suitable for publication in *PLoS ONE*. We look forward to hearing from you.

Sincerely,

Nadia Shobnam

National Institute of Allergy and Infectious Diseases (NIAID)

Email: [nadia.shobnam@nih.gov](mailto:nadia.shobnam@nih.gov)

Phone: 302-345-1118
